# The anatomic feasibility of thoracic branched endoprosthesis in the treatment of blunt thoracic aortic injury

**DOI:** 10.3389/fsurg.2025.1667618

**Published:** 2026-01-22

**Authors:** Anna Rogalska, Ashley Flinn-Patterson, Maria Navarro, Stephanie Combs, Theodore Hart, Marlin Causey

**Affiliations:** Brooke Army Medical Center, San Antonio, TX, United States

**Keywords:** vascular, surgery, endovascular, trauma, thoracic, aortic

## Abstract

**Introduction:**

Blunt thoracic aortic injury (BTAI) is one of the leading causes of death among trauma patients who sustain high impact thoracic trauma with rapid deceleration. Thoracic endovascular aortic repair (TEVAR) is indicated in high grade injuries and requires a management strategy for the left subclavian artery (LSA). Gore TAG thoracic branch endoprosthesis (TBE) is a newly approved TEVAR device for to maintain LSA patency utilizing a side branch with reported use in acute indications. The anatomic suitability of this device for a population of BTAI patients and optimal inventory for off-the-shelf emergent repairs has not been reported.

**Methods:**

A retrospective analysis of 66 patients admitted to a Level 1 Trauma Center who sustained BTAI between January 2011 and December 2023 and underwent TEVAR was performed. Computed tomography imaging was analyzed on all patients to determine the suitability for repair according to instructions for use (IFU) criteria of the manufacturer.

**Results:**

The distance between the LSA and the injury was less than 2 cm in 59% of patients, representing a possible indication for TBE. The average injury distance in this cohort was 9 mm from the LSA, with 82% of these patients meeting IFU requirements for TBE and 18% requiring standard TEVAR. For patients who met TBE graft requirements, 28 mm, 31 mm, and 34 mm aortic components fit 76% of patients and 10 mm and 12 mm subclavian branches fit 87% of patients. Patients who did not meet IFU requirements for TBE were sized for standard TEVAR with 26 mm, 31 mm, and 34 mm grafts treating 66% of patients.

**Conclusions:**

This study demonstrates key anatomic considerations and models the suitability and optimal TBE inventory drawn from a real-world population of BTAI patients. Despite a vast device catalog for TEVAR, this study demonstrates that carrying three TBE aortic components, two TBE subclavian components, and three standard TEVAR sizes would treat 71% of BTAI patients.

## 1 Introduction

Blunt thoracic aortic injury (BTAI) is one of the leading causes of death among trauma patients who sustain multiple blunt injuries (1). Over the last 20 years, thoracic endovascular aortic repair (TEVAR) has revolutionized treatment of BTAI and is now the standard for thoracic aortic repair given its reduced morbidity and mortality across multiple indications (2). A systematic review of patients who underwent endovascular intervention in the setting of aortic transection, the most severe traumatic aortic injury, demonstrated a mortality rate of 7.6% which is half than open intervention (3). This data is the foundation for the Clinical Guidelines of the Society of Vascular Surgery which recommend TEVAR for significant thoracic aortic injuries that are anatomically suitable regardless of age (4). Recent innovations in TEVAR device design have enabled preservation of left subclavian artery flow which is the focus of our present study.

Up to 60% of BTAI occurs at the isthmus of the aorta, which is just distal to the left subclavian artery (LSA) near the site of the ductus arteriosus. This mobile anatomic location undergoes strain and deceleration injury in traumatic events causing intimal tears, hematomas, pseudoaneurysms and even full thickness transection manifesting as rupture (5). Evaluation based on the degree of injury using a staging system, as well as the anatomical location, plays a role in interventional planning. Given that one of the most common injury locations is near the LSA, 40% of BTAI patients will require LSA coverage when using a standard TEVAR approach (6). In these instances, LSA coverage is necessary to obtain an adequate proximal landing zone. Coverage of the LSA is not benign; it is associated with complications such as stroke, spinal cord ischemia, paraplegia and vertebrobasilar and left arm ischemia (7). Studies report stroke incidence after TEVAR as 3.8%–6.3%, while upper extremity ischemia incidence can be 12%–20% (8). Current guidelines recommend preoperative revascularization of the LSA for zone 2 TEVAR (6). This can cause additional planning challenges in trauma patients who may require more urgent intervention and would benefit from a single stage procedure.

The current management paradigm of BTAI includes allowing time for patient stabilization and upfront treatment of other severe traumatic injuries, allowing the ability to appropriately plan and prepare TEVAR including consideration for the preservation of left upper extremity circulation (9, 10). In 2022, a thoracic branched endoprosthesis (TBE; Gore, Flagstaff, AZ, USA) device was approved by the United States Food and Drug Administration (FDA) representing a new off-the-shelf option for thoracic aortic repair and in order to preserve flow to the LSA (11). The IFU specifications include parameters for adequate access, a non-dissected proximal aortic landing zone with inner diameter 16–42 mm and sufficient proximal coverage of 15–36 mm allowing for 2–4 cm distance between branch vessels depending on the size of aortic component selected, target branch vessel inner diameters 6–18 mm with 2.5–3.0 cm length to the vertebral depending on the size of the side branch portal selected, and suitable quality distal landing zone with inner diameter 16–42 mm with at least 2 cm from the celiac artery measured on the outer curve (11).

The main objectives of the study were to first determine the anatomic suitability of the TBE device applied in a real-world population of patients treated at a Level 1 Trauma Center through retrospective analysis of previously treated cases through comparison of measurements to the IFU criteria. Acknowledging a proportion of BTAI patients was previously treated with TEVAR and LSA coverage before the TBE device, the study seeks to define the proportion of these patients that qualify anatomically for a TBE in retrospect. Secondarily, the study aims to use the anatomic distribution of the real-world population to describe the required inventory components when adopting this new approach by sizing this real-world cohort for devices to model what is required for off-the-shelf treatment.

## 2 Methods

This is a retrospective analysis of 66 patients admitted to a Level 1 Trauma Center who sustained BTAI between January 2011 and December 2023. The BTAI database was queried for patients receiving TEVAR during this time frame to avoid gathering data on patients who would not have been surgically treated. All non-traumatic aneurysms and dissections were excluded. We utilized computed tomography angiography imaging to define each patient's anatomy, determine options for aortic repair, and identify commonly used inventory for adequate planning. Detailed aortic and subclavian measurements were obtained using 3-D reconstruction of computed tomography images using TeraRecon (Durham, NC, USA), technology to ensure visualization of anatomic properties. All options for thoracic repair were within approved instruction for use (IFU). Each patient was fitted for either a TBE device, which included an aortic component and a subclavian component, or a standard TEVAR graft based on their anatomy. Patients who were possible TBE device candidates with an aortic injury <2 cm from the LSA were sized based on aortic and subclavian measurements using centerline and/or greater curve of artery. Patients with an injury greater than 2 cm from the LSA are suitably treated with standard TEVAR without LSA coverage given the sufficient proximal seal. Anatomic measurement values including the distance from LSA to branch vessels and aortic injury, aortic and subclavian diameter measurements, were calculated to fit each patient into their appropriate graft size utilizing both centerline and greater curve measurement techniques. Institutional review board ethical approval was obtained for this study. Authors AR, AF, MN, TH, MC performed measurements for the study. Each patient was measured by at least two authors to ensure agreement in sizing.

Demographic and anatomic measurements were summarized using descriptive statistics. Group comparisons between TBE-eligible and non-eligible patients were performed with chi-square tests for categorical variables and Student's *t*-tests for continuous variables. Statistical analyses were conducted with significance set at *p* < 0.05.

## 3 Results

**Figure 1:**
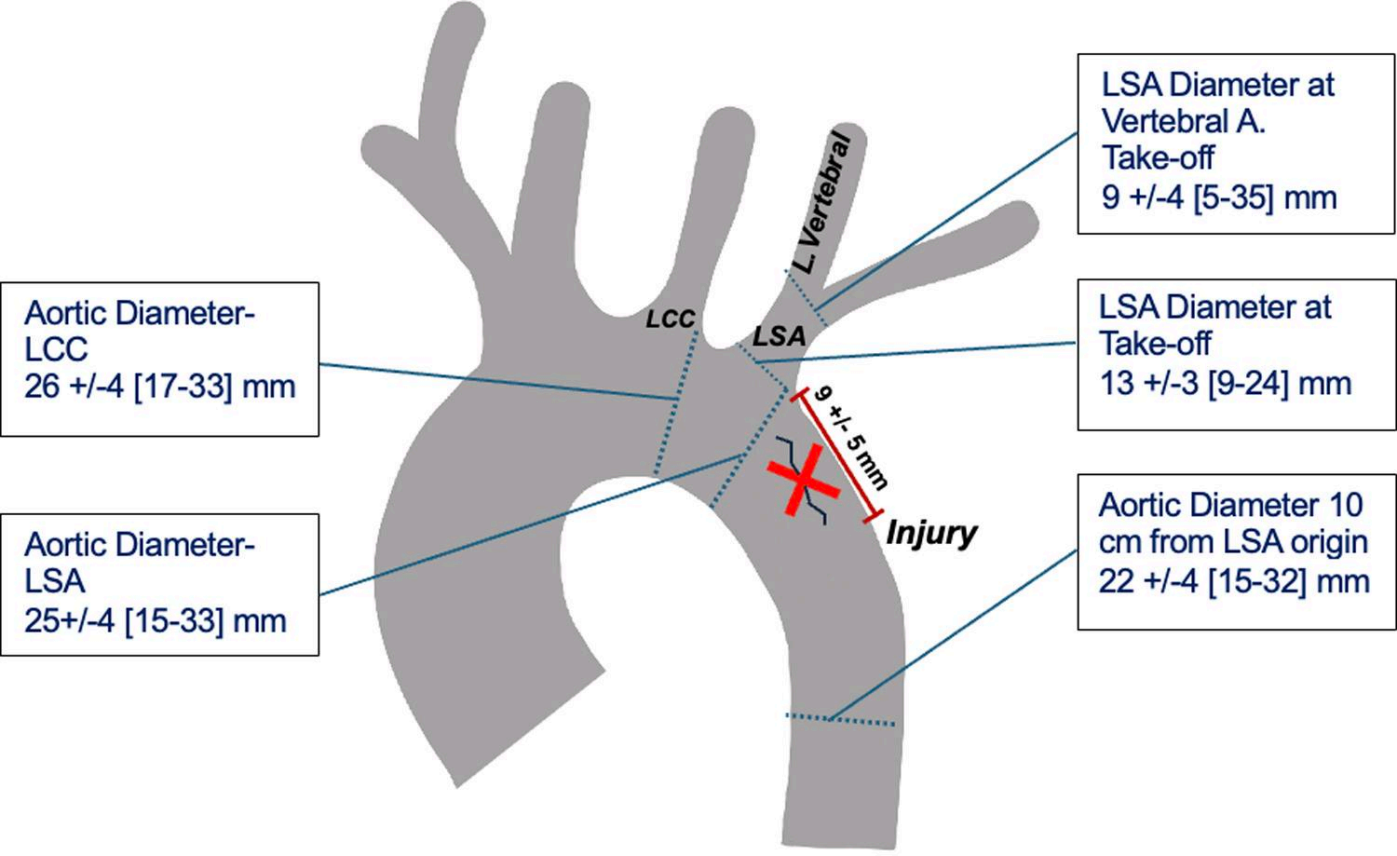
Anatomic measurement averages of TBE candidates.

Among the 66 patients meeting inclusion criteria, 76% were male with a mean age of 45 (±17 years). All patients evaluated had sufficient external iliac artery diameter for sheath introduction. Two patients were disqualified for aortic origin of the left vertebral artery and one patient had aberrant origin of the right subclavian. In 68% of patients, the distance between the LSA and the injury was less than 2 cm, which was the initial qualification considered for TBE candidacy (Table 1). Based on measurements of additional IFU criteria including aortic and subclavian diameters and branch vessel distances, 82% of patients with an injury distance <2 cm from the LSA would have been candidates for a TBE device; this represented56% of the total BTAI cohort examined when accounting for the additional anatomic anomalies. The remaining 18% of the patients did qualify for a TBE device based on IFU criteria and require a standard TEVAR graft. The average injury distance from LSA measured was 9 ± 5 mm in TBE candidates (Figure 1). The cohort of patients qualified for TBE mirrored the overall cohort, 77% male with a mean age of 43 (±15), and there was no significant difference in gender, age, or other difference in arch branch candidacy (*p* = not significant).

**Table 1:** Delineation of candidacy of TBE repair based on arch anatomy in patients with an aortic injury .

Demographics			
	*N*		66
	Age (mean ± SD)		45 (±17)
	Gender	Male, *n*	50
		Female, *n*	16
Aortic injury to LSA <2 cm, *n*			
	Yes		45
	No		21
Aortic candidate for TBE, *n*			
	Yes		
	Centerline		13
	Greater curve		24
	Both		24
	No		3
LSA candidate for TBE, *n*			
	Yes		
	Centerline		29
	Greater curve		8
	Both		8
	No		3
Anatomic variant, *n*			
	Aberrant right subclavian artery		1
	Vertebral originating from aorta		2
Total patients, *n*			
	TBE (%)		37 (56)
	TEVAR (%)		29 (44)

**Figure 2:**
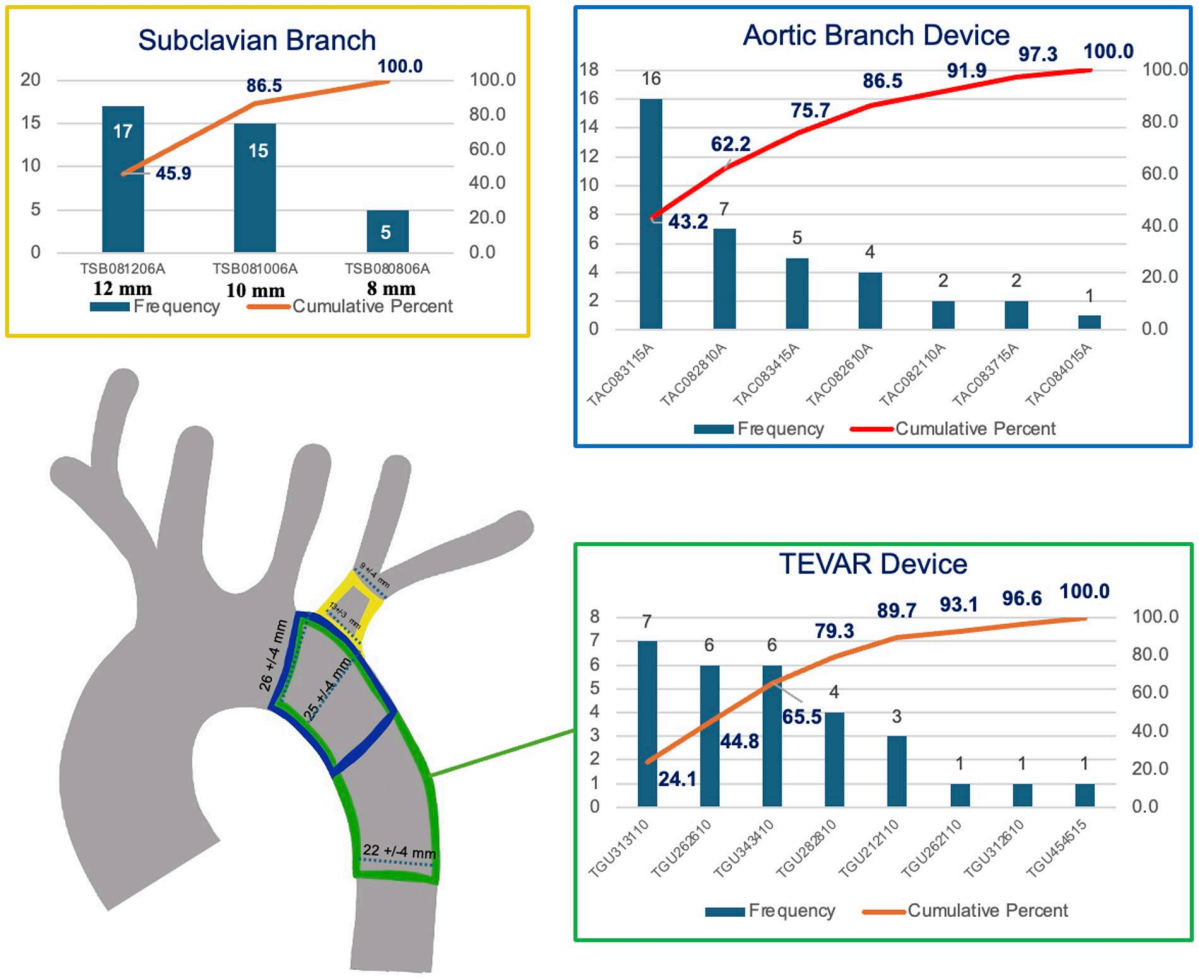
Sizing based off currently available TBE grafts for BTAI.

Examining the 37 patients in our cohort suitable for TBE, sizes 28 mm, 31 mm, and 34 mm diameter aortic components fit 76% of patients with subclavian branches of 10 and 12 mm, treating 87% of patients (Figure 2). Patients not candidates for the TBE device met sizing criteria for standard TEVAR by the same manufacturer with 66% percent of patients treated with 26 mm, 31 mm, and 34 mm grafts. The remaining 34% were treated with TEVAR sizes outside these top three devices, which included sizes 21 mm, 28 mm and 45 mm. Our population did not utilize any 37 mm or 40 mm grafts, but those options exist in the inventory as well.

## 4 Discussion

The main findings of the study were that approximately two thirds of a real-world population of BTAI patients had an injury location appropriate for TBE application. After excluding aberrant anatomic cases and performing sizing utilizing centerline and greater curve techniques, more than three quarters of this population met IFU criteria for a TBE device. Taken together, just over half of the examined cohort was appropriate for TBE anatomically. These findings suggest that institutions should make practical consideration of an inventory of devices to support TBE as well as standard TEVAR. Examination of the aortic and branch component sizing across our cohort demonstrated that an inventory of three aortic components (28 mm, 31 mm, 34 mm) and two branch components (10 mm and 12 mm) allows for off-the-shelf treatment in the vast majority of TBE cases. The sizing in standard TEVAR demonstrated slightly more variation with two thirds of patients being treated with a 26 mm, 31 mm, or 34 mm device.

Current management of BTAI patients depends on the overall grade of injury and the patient presentation. Computed tomography with intravenous contrast is used for diagnosis (12, 13). Grade I injuries, or intimal tears, do not require intervention and medical management with impulse control is recommended. For grade III (pseudoaneurysm) and grade IV (rupture) aortic lesions, the clinical practice guidelines recommend TEVAR intervention in addition to impulse control, with grade IV requiring the most emergent or urgent repair (5). For TEVAR, appropriate anatomic sizing is performed. The goal is to provide adequate injury coverage while limiting coverage length. In many cases, LSA coverage optimizes a proximal landing zone, but unfortunately may compromise arm perfusion.

In 2022, a thoracic branched endoprosthesis (TBE) graft was designed and approved for use for thoracic aortic repair. This device prevents LSA coverage, while covering the area of injury. This study demonstrates which branched and standard TEVAR graft sizes would optimally treat 71% of patients who sustain BTAI and require intervention. Within a trauma center, readily available off-the-shelf TBE devices can be a valuable management option for BTAI patients. Not only are patients treated with a single-stage procedure, but the patients also do not have to be subject to the possible complications associated with LSA coverage, especially when other injuries limit clinical exam. TBE is new on the market and has not yet been recommended as the gold standard for clinical practice given a lack of long term follow up (5). However, it has been evaluated for its safety profile and thus far has proven to have a low rate of complications in early studies using smaller trauma populations that underwent TBE and case series including 6 and 9 patients (14–16). Larger cohort studies with long-term data are still pending for these devices, but thus far institutional experience has demonstrated effective results in the treatment of traumatic aortic injuries. Given this information, patients who sustain BTAI should be evaluated on whether they are a candidate for TBE repair. If they are not a TBE candidate, they should undergo standard TEVAR. In instances where the aortic injury is greater than 2 cm from the LSA or an anatomical anomaly is present (44% of patients in our study) TEVAR devices should be utilized rather than TBE. However, 56% of patients in our study would have met IFU standards and would have potentially benefited from the TBE device.

While investigating whether individual patients fit the standard TEVAR or TBE devices is vital, it is also pertinent to apply this knowledge at a systems level. This information is useful when planning urgent and emergent cases using off-the-shelf devices at trauma centers. With many sizes available for stocking, our practice is to maintain an inventory inclusive of the three most common grafts utilized to be able to offer this minimally invasive option to as many patients as possible who could potentially benefit. In today's market, manufacturing companies Gore & Associates, Bolton Medical, Inc., Medtronic, and Cook Medical are four FDA-approved options for thoracic sizes for TEVAR in the United States. The CastorTM endograft (Lombard Medical, Didcot, United Kingdom) is not FDA approved, but represents a similar LSA-branched thoracic endograft which is very popular in China and Europe and utilized for acute aortic syndromes (17). Graft sizing is expansive to allow coverage for the outliers of aortic anatomy. Current devices on the market for TEVAR range from 21 to 46 mm to fit aortic diameters of 16–42 mm allowing for oversizing (18). As far as TBE devices, the manufacturing company Gore & Associates provides similar aortic sizing of 21–46 mm and is currently the only FDA-approved manufacturer for TBE (11).

Inventory is critical in the preoperative planning for aortic repair. In centers that have TEVAR capabilities for trauma, off-the-shelf devices are essential, and this data serves to assist surgeons in making decisions on which grafts are optimal to maintain on their shelves. To anatomically treat, with optimal graft sizing, this study suggests having 28 mm, 31 mm, and 34 mm aortic components, 10 mm and 12 mm subclavian components, and the 26 mm, 31 mm, and 34 mm TEVAR sizes allows optimal anatomic treatment of 71% of patients in our real-world cohort.

A limitation in our study is that it is a retrospective analysis as opposed to a prospective analysis. This study did not look at cost effectiveness of the treatment, acknowledging that TBE grafts are more expensive; however, there are costs associated with concomitant or subsequent open revascularization including added operative time for a hybrid debranching when applicable. Our data analysis is also limited by sample size and to only those patients who were treated with repair and did not include grade I and II aortic lesions, which we feel appropriate for this type of analysis given that treatment of these injuries is largely nonoperative. Our study utilizing only Gore products for TEVAR anatomic sizing, which may not align with other companies.

## 5 Conclusions

This study evaluates a real-world population of trauma patients for the relevant anatomic considerations in applying TBE, including injury location, aortic branch pattern, and vessel diameters. There is no “one size fits all” in BTAI and TBE will be anatomically applicable in just over half of cases. Maintaining a device inventory of the three most utilized aortic components, the two most utilized branch components, and the three most utilized standard TEVAR grafts allows treatment of 71% of BTAI patients in an off-the-shelf manner.

## Data Availability

The original contributions presented in the study are included in the article/Supplementary Material, further inquiries can be directed to the corresponding author.
